# Designing main-group catalysts for low-temperature methane combustion by ozone

**DOI:** 10.1038/s41467-023-39541-y

**Published:** 2023-07-03

**Authors:** Shunsaku Yasumura, Kenichiro Saita, Takumi Miyakage, Ken Nagai, Kenichi Kon, Takashi Toyao, Zen Maeno, Tetsuya Taketsugu, Ken-ichi Shimizu

**Affiliations:** 1 Institute for Catalysis, Hokkaido University, N-21 W-10, Sapporo, Hokkaido 001-0021 Japan; 2 Department of Chemistry, Faculty of Science, Hokkaido University, Sapporo, Hokkaido 060-0810 Japan; 3 School of Advanced Engineering, Kogakuin University, Tokyo, 192-0015 Japan; 4 Institute for Chemical Reaction Design and Discovery (WPI-ICReDD), Hokkaido University, Sapporo, Hokkaido 001-0021 Japan

**Keywords:** Heterogeneous catalysis, Catalytic mechanisms, Computational chemistry

## Abstract

The catalytic combustion of methane at a low temperature is becoming increasingly key to controlling unburned CH_4_ emissions from natural gas vehicles and power plants, although the low activity of benchmark platinum-group-metal catalysts hinders its broad application. Based on automated reaction route mapping, we explore main-group elements catalysts containing Si and Al for low-temperature CH_4_ combustion with ozone. Computational screening of the active site predicts that strong Brønsted acid sites are promising for methane combustion. We experimentally demonstrate that catalysts containing strong Bronsted acid sites exhibit improved CH_4_ conversion at 250 °C, correlating with the theoretical predictions. The main-group catalyst (proton-type beta zeolite) delivered a reaction rate that is 442 times higher than that of a benchmark catalyst (5 wt% Pd-loaded Al_2_O_3_) at 190 °C and exhibits higher tolerance to steam and SO_2_. Our strategy demonstrates the rational design of earth-abundant catalysts based on automated reaction route mapping.

## Abstract (web summary)

Automated reaction route mapping is used to design catalysts for low-temperature CH_4_ combustion with ozone. A suitable proton-type zeolite catalyst with Brønsted acid sites was predicted and shown to have superior performance in CH_4_ combustion.

## Introduction

The past decades have witnessed the widespread utilization of natural gas as a clean fuel for vehicles and power plants. The catalytic combustion of methane (CH_4_) into carbon dioxide (CO_2_) is becoming an increasingly valuable strategy for addressing the emissions of unburned CH_4_, which exerts a greenhouse gas effect that is 22 times higher than that of CO_2_^1–3^. Different types of heterogeneous catalysts, such as platinum-group-metal (PGM-)^4–7^ and metal-oxide-based catalysts^8–10^, have been reported. Among them, PGM-based catalysts, such as Pd- and Pt-loaded Al_2_O_3_, exhibited the highest catalytic activities^11^. However, the Pd-based catalysts suffer from high operating temperatures (>500 °C) under humidity conditions, as well as irreversible deactivation by sulfation during the co-feeding of steam and SO_2_^2,12–14^. Moreover, large amounts of PGMs (200–266 g) must be utilized to achieve the combustion of CH_4_ in a natural-gas-fueled heavy-duty vehicle^15^. Additionally, the mining and purification of PGMs extensively impact the environment (the productions of 1 kg each of Pt and Pd generate 12,500 and 3880 kg of CO_2_ equivalents, respectively). Conversely, the production of main-group elements generates significantly lower CO_2_ equivalents (e.g., 8.2 kg of Al)^16,17^. Thus, it is highly desirable (economically and ecologically) to develop main-group catalysts that can function at <200 °C in the co-presence of steam and SO_2_.

Conventional catalyst screening, which is based on trial-and-error experiments, may not yield discontinuous discoveries, such as the main-group-facilitated catalytic combustion of CH_4_ at low temperatures. The computational reaction route mapping of the unexplored chemical reaction space can benefit the discovery of different catalytic reactions^18–23^. Generally, the computations of the elementary steps in combustion reactions are considered challenging because of the abundant intermediates and products that exhibit similar formation energies and activation barriers (*Ea*)^24^. To comprehensively explore the various reaction routes, density functional theory (DFT)-based automated methods for predicting reaction pathways are promising because they link the theoretical prediction to the practical designs of catalysts^25–30^. Maeda et al. developed an efficient automated path-searching method, namely the artificial force-induced reaction (AFIR) method, which involves pressing the atoms in given reactant molecules together by applying artificial force to form new structures (products) and assigning their transition states (TS)^30–41^. Via AFIR, they elucidated the entire reaction pathways of uncatalyzed reactions^32,37,39,40^. The automated reaction route mapping of heterogeneous catalysis systems is still formidable owing to the complexity of the surface reactions on solid materials, where the adsorption/desorption of the reactants and products, diffusion/migration of the adsorbates, and bond rearrangements proceed simultaneously^35,36,41^.

Ozone (O_3_), a strong oxidant^42^, is generated onsite by a commercial ozonizer. O_3_ has been employed to enhance the catalytic performance of the gas-phase combustion of volatile organic compounds, including toluene^43–45^, acetone^46,47^, and benzene^48,49^. Regarding the combustion of CH_4_ with O_3_^50–52^, zeolite-based catalysts, such as Pd^53^−, Fe^54^−, Co^55^−, and proton^56^-type zeolites, have demonstrated efficiencies at low temperatures. However, the reported studies only considered the catalytic performance; thus, the strategy for designing the catalysts based on the detailed mechanism and elementary steps must still need to be addressed.

**Fig. 1:**
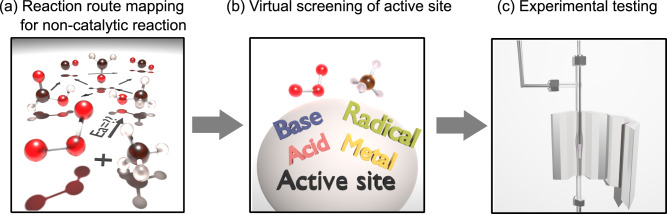
Rational design concept for catalytic combustion of CH_4_ with O_3_. **a** Employing single-component (SC)-AFIR, CH_4_ combustion with O_3_ was comprehensively explored to determine the key intermediates and elementary steps. **b** Different active sites were evaluated regarding the decrease in *E*_*a*_ of the key elementary step. **c** Heterogeneous catalyst comprising the predicted active site was tested experimentally.

Herein, based on a computational design concept employing the AFIR method, we report a main-group catalyst for driving catalytic combustion of CH_4_ with O_3_ at low temperatures. First, we explored the CH_4_ + O_3_ reaction network toward generating CO_2_ (Fig. 1a), confirming that the formation of methanol (CH_3_OH), CH_4_ + O_3_ → CH_3_OH + O_2_, was the rate-determining step (RDS) of CH_4_ combustion. Thereafter, we performed the virtual screening of the active sites for RDS to propose the following concept: stronger Brønsted acid sites (BASs) exhibit higher catalytic activities (Fig. 1b). This concept was experimentally verified via CH_4_ combustion tests employing O_3_ at 250 °C in the presence of different BAS catalysts exhibiting different acid strengths (Fig. 1c). Finally, we demonstrated that a proton-type beta zeolite with Si/Al = 8.5 (Hß8.5) exhibited a reaction rate that was three orders of magnitude higher than that of a PGM-based benchmark catalyst, 5 wt% Pd-loaded Al_2_O_3_ (Pd5Al_2_O_3_). The developed catalyst exhibited very high resistance to steam and SO_2_ poisoning during the 170-h reaction test.

## Results

### Computation of the reaction pathways toward CH_4_ combustion by O_3_

**Fig. 2:**
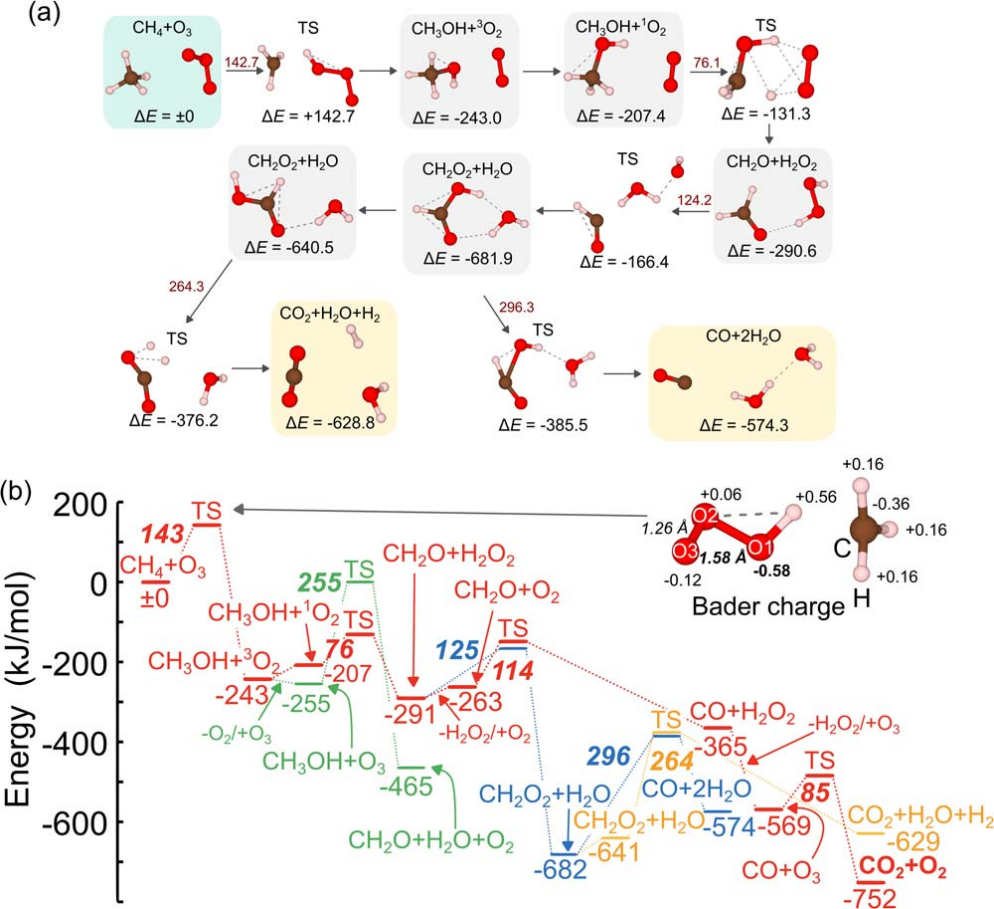
Result of reaction route mapping for CH_4_ + O_3_ reaction without active sites. **a** Calculated reaction pathway of CH_4_ + O_3_, as well as the values of relative energies (Δ*E*s). The values written in dark red represent *E*_*a*_. **b** Energy profile of CH_4_ combustion to yield CO_2_. The reaction path shown by the red lines is the most plausible for CO_2_ formation. The result of the Bader charge analyses of the TS structures of CH_4_ + O_3_ and CH_3_OH + O_3_ is shown together. Δ*E*s are provided under each bar, and the *E*_*a*_ are described employing the bold italic style (Unit: kJ/mol).

We explored the reaction pathway of CH_4_ and O_3_ (CH_4_ + O_3_) via SC-AFIR, which was an automated method for searching for reaction paths, as implemented in the GRRM program. Employing this method, the reaction routes of the non-catalytic oxidation of CH_4_ into CO_2_ by O_3_ are automatically mapped. Figure 2a shows the reaction pathways with the corresponding values of their relative energies (Δ*E*) and *Ea*. In the first reaction, CH_4_ is oxidized by O_3_ to yield CH_3_OH and O_2_ with strong exothermicity (243.0 kJ/mol). In the TS structure, one O atom of O_3_ extracts one H atom of CH_4_ to yield CH_3_ and OOOH fragments, where the evaluated *E*_*a*_ is 142.7 kJ/mol. This value is comparable to the reported experimental value for gas-phase CH_4_ combustion by O_3_ (148 kJ/mol)^57^. Next, the reactivity of CH_3_OH with O_3_ is assessed by exploring the reaction pathway via the SC-AFIR method (see Supplementary Fig. S1). Although CH_3_OH is oxidized into formaldehyde (CH_2_O), H_2_O, and O_2_ by O_3_, CH_3_OH + O_3_ $\to$ CH_2_O + H_2_O + O_2_, via an exothermic reaction (210.1 kJ/mol), the process requires a very high *E*_*a*_ (255.1 kJ/mol). Alternatively, the oxidation of CH_3_OH by O_2_ produces CH_2_O and H_2_O_2_, CH_3_OH + O_2_ $\to$ CH_2_O + H_2_O_2_, via a low *E*_*a*_ of 76.1 kJ/mol (Fig. 2a). The subsequent oxidation of CH_2_O by H_2_O_2_ yields formic acid (CH_2_O_2_) and H_2_O, CH_2_O + H_2_O_2_ $\to$ CH_2_O_2_ + H_2_O, with an *E*_*a*_ of 124.2 kJ/mol. The decomposition of the produced CH_2_O_2_ yields CO_2_ and H_2_, CH_2_O_2_ $\to$ CO_2_ + H_2_, or CO and H_2_O molecules, CH_2_O_2_ $\to$ CO + H_2_O. However, these decomposition processes require high *E*_*a*_ to produce CO_2_ and CO (264.3 and 296.3 kJ/mol, respectively) because of the high stability of CH_2_O_2_. As an alternative reaction path, we explored the oxidation of CH_2_O by O_2_, which was abundantly present in the practical systems, via SC-AFIR (see Supplementary Fig. S2). Thus, the oxidation of CH_2_O by O_2_ represents a facile process for producing CO and H_2_O_2_ via an *E*_*a*_ of 113.7 kJ/mol. The CO was oxidized into CO_2_ + O_2_ by O_3_ through an *E*_*a*_ of 84.9 kJ/mol (see Supplementary Fig. S3). For comparison, Nitrous oxide (N_2_O) and H_2_O_2_ were assessed as alternative oxidants to oxidize CH_4_ into CH_3_OH (see Supplementary Fig. S4). The evaluated *E*_*a*_ of the CH_4_ + N_2_O and CH_4_ + H_2_O_2_ reactions are 269 and 177 kJ/mol, respectively, indicating that O_3_ is the most efficient oxidant for producing CH_3_OH.

Employing the explored reaction pathways (Fig. 2b), the reaction, CH_4_ + O_3_ $\to$ CH_3_OH + O_2_, was determined as the crucial process, with the highest *E*_*a*_ in the CH_4_ oxidation reaction (the RDS). To further elucidate the TS structure of this reaction, Bader charge analysis was performed to investigate the distribution of charge on each atom in the TS structure (Fig. 2b). The total atomic charges in the CH_3_ fragment are almost neutral (+0.12), indicating that it is a radical-like fragment. Regarding the OOOH fragment, the structure is divided into two parts: (I) the part comprising the H and O atoms that are closer to the CH_3_ fragment (denoted as O1) and (II) that comprising the other two O atoms (denoted as O2 and O3). In the former part, the determined atomic charges of the H and O1 atoms are +0.56 and −0.58, respectively, while those of the O2 and O3 atoms in the latter part are +0.06 and −0.12, respectively. This charge distribution indicates that the OOOH fragment comprises a OH radical and O_2_ molecular species.

### Virtual screening of catalytic sites for the reaction of CH_4_ + O_3_ to produce CH_3_OH + O_2_

**Fig. 3:**
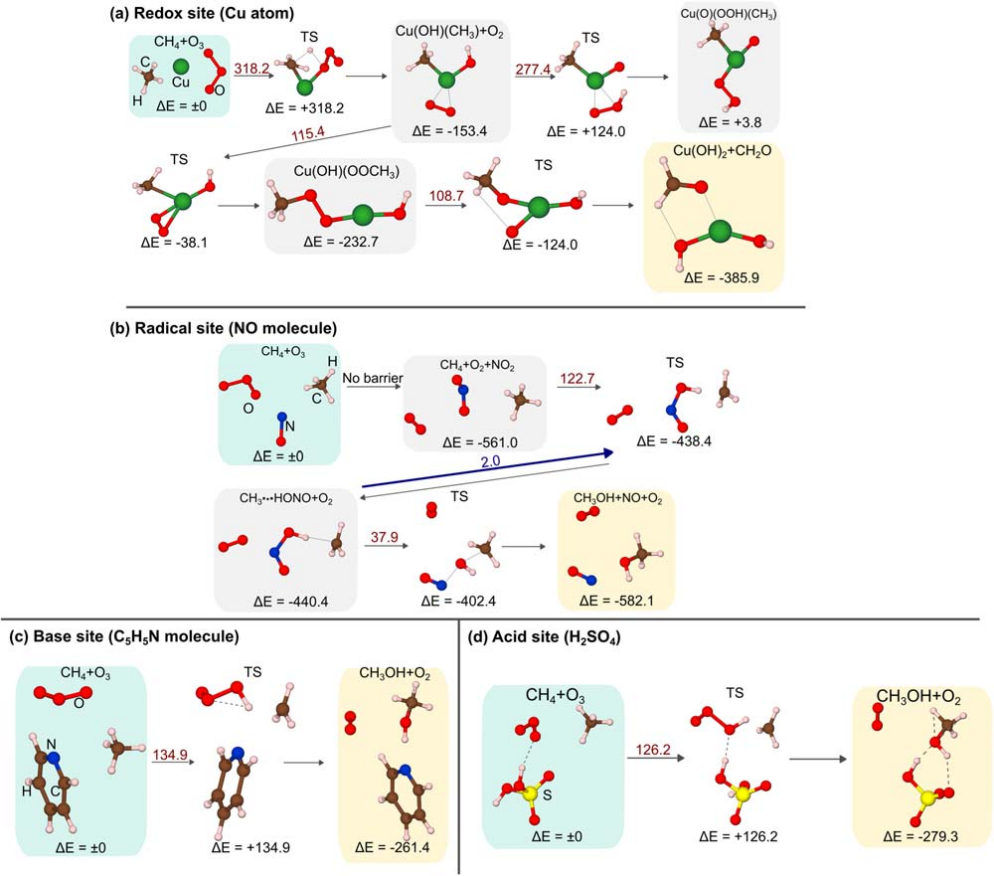
Result of reaction route mapping for CH_4_ + O_3_ reaction on model active sites. Calculated reaction pathways of CH_4_ + O_3_ on **a** Cu(0) atom, **b** NO molecule, **c** C_5_H_5_N molecule, and **d** H_2_SO_4_ molecule. The values of Δ*E* and the *E*_*a*_ (dark red) are shown together (Unit: kJ/mol).

The oxidation of CH_4_ by O_3_ into CH_3_OH and O_2_ was determined as the key reaction during CH_4_ combustion (Computation of the reaction pathways toward CH4 combustion by O_3_). To conduct the virtual screening of the catalytically active sites that effectively decrease the *Ea*, we carried out SC-AFIR calculations for the CH_4_ + O_3_ reaction on the following model active sites: (a) a Cu(0) atom as a redox site, (b) an NO molecule as a radical species, (c) pyridine (C_5_H_5_N) as a Brønsted and Lewis base site, and (d) sulfuric acid (H_2_SO_4_) as a Brønsted acid site (Fig. 3). Lewis acid site is not considered here because it is known that Lewis acid sites are rapidly deactivated in the presence of H_2_O and SO_2_, which are abundantly contained in the exhaust gases and produced by the CH_4_ combustion reaction. Figure 3a shows the reaction path of CH_4_ + O_3_ over Cu(0). First, the C–H bond of CH_4_ is cleaved by the O atom of O_3_ over the Cu atom to yield OH and CH_3_ groups on the Cu(II) cation (Cu(OH)(CH_3_)), as well as adsorbed O_2_ molecules through a high *E*_*a*_ (318.2 kJ/mol). Subsequently, the adsorbed O_2_ molecule interacts with the neighboring CH_3_ group to form CH_3_OO species on the Cu(II) cation (Cu(OH)(CH_3_OO)) via an *E*_*a*_ of 115.4 kJ/mol, while the extraction of the H atom of the OH group (Cu(O)(OOH)(CH_3_)) is determined as an unfavorable path. Finally, the Cu(OH)_2_ species and CH_2_O are produced with a moderate barrier (108.7 kJ), although the Cu(II) cation was not reduced back into the Cu(0) atom. The maximum barrier was higher than that of the uncatalyzed reaction (142.7 kJ/mol). Hence, the Cu(0) atom was not a suitable catalyst for the CH_4_ + O_3_ reaction. Further, the NO molecules as a representative radical site reacted with O_3_ to yield O_2_ and NO_2_, where the H atom of CH_4_ was subsequently extracted to yield the CH_3_ radical species that were bound to the nitrous acid (HONO) species (Fig. 3b). Although the evaluated *E*_*a*_ of this step was relatively low (122.7 kJ/mol), that of the reverse reaction (CH_3_• + HONO → CH_4_ + NO_2_) was very low (2.0 kJ/mol). Additionally, the reaction, NO_2_ + CH_4_, to yield CH_3_OH + O_2_ shows only a low exothermicity (−21.1 kJ/mol). These results indicate that NO is also an inefficient catalyst for the CH_4_ + O_3_ reaction. To compare the result above with more realistic models, the reaction route mappings on the Cu metal cluster and FeO species in ZSM-5 zeolite were carried out only for the first step of CH_4_ activation (supplementally Fig. S5). Note that the CH_4_ activation on FeO species in ZSM-5 zeolite is known to proceed via the formation of CH_3_ radical, similar to the case of the NO molecule in Fig. 3b^58^. The results indicate that, although *E*_*a*_ for CH_4_ are different, the tendency of each active site is similar (high *E*_*a*_ of reducing back and low *E*_*a*_ of reverse-reaction for metal and radical sites, respectively). In the case of C_5_H_5_N as a base site, O_3_ slightly interacted with the basic site (the N atom) of C_5_H_5_N before reacting with CH_4_; thus, O_3_ was not decomposed by the active site (Fig. 3c). Thereafter, CH_3_OH was produced through a similar TS structure to the gas-phase one, and the product, which was weakly bound to the base site, was slightly more stable than that in the gas phase (Δ*E* = − 264 kJ (on the base site) vs −243 kJ (in the gas phase)), while their *E*_*a*_ were comparable (*E*_*a*_ = 134.9 vs 142.7 kJ/mol). Finally, H_2_SO_4_ was assessed as an acid site. CH_3_OH was produced via an *E*_*a*_ of 126.2 kJ/mol, which was lower than that of the gas-phase reaction (142.7 kJ/mol), with very high exothermicity (Δ*E* = − 279.3 kJ/mol; Fig. 3d). As representative PGMs, Pd atom was also assessed as a potential active site for CH_4_ + O_3_ reaction (Supplementary Fig. S6). The result shows that the highest activation barrier in the reaction coordinates toward CH_2_O is 109.3 kJ/mol. Since Pd-based catalysts are known to be efficient catalysts for CH_4_ combustion by O_2_, our result agrees with the previous reports^3,53,57,59^. Thus, the activity of Pd-based catalysts (Pd-loaded Al_2_O_3_ and Pd-exchanged zeolite) for CH_4_ + O_3_ reaction will be experimentally tested later. Consequently, we predicted that BASs were the most effective among the virtually screened active sites for CH_4_ + O_3_. In the next section, we discussed the preferable property of BAS, as well as the detailed mechanism of decreasing *E*_*a*_.

### Promotion effect of BAS on the CH_4_ + O_3_ reaction

We investigated the effect of the acid strength of BASs of the mineral acids on the *E*_*a*_ of CH_4_ + O_3_. H_2_SO_4_, perchloric acid (HClO_4_), nitric acid (HNO_3_), and phosphoric acid (H_3_PO_4_) were evaluated as BASs exhibiting different acidities. The initial structure (IS), final structure (FS), and TS are shown in Supplementary Fig. S7. The *E*_*a*_ of the strong acids (128.4 and 126.2 kJ/mol for HClO_4_ and H_2_SO_4_, respectively) are lower than that of the uncatalyzed reaction (142.7 kJ/mol), which is close to the value for a weak acid (142.2 and 138.8 kJ/mol for H_3_PO_4_ and HNO_3_, respectively).

**Fig. 4:**
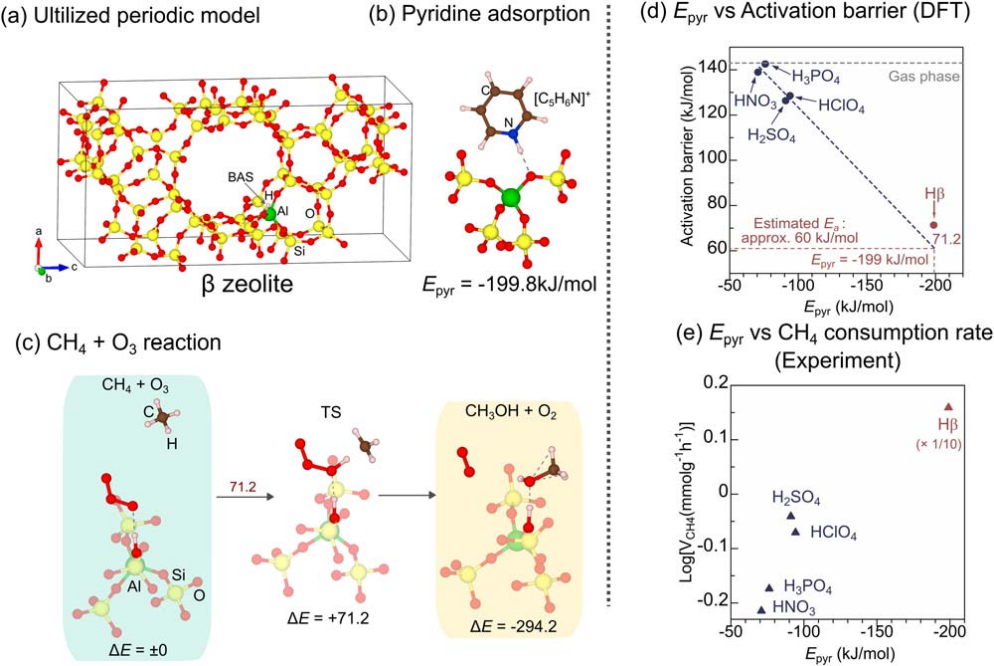
Theoretical and experimental evaluation for the effect of acid sites on CH_4_ + O_3_ reaction. **a** Employed periodic model of the ß zeolite. **b** Structure of the adsorption of C_5_H_5_N on BAS of ß zeolite. **c** TS calculations of the CH_4_ + O_3_ reaction on BAS of the ß zeolite. Δ*E* is reported in the kJ/mol unit. *E*_*a*_ is shown in dark red. **d** Plot of *E*_*a*_ of the CH_4_ + O_3_ reaction as a function of the C_5_H_5_N-stabilization energy (*E*_pyr_) of the acids. **e** CH_4_ consumption rate of the samples in 0.1% CH_4_ + 0.7% O_3_ at 250 °C (He balance, total flow: 100 ml/min) as a function of *E*_pyr_.

**Fig. 5:**
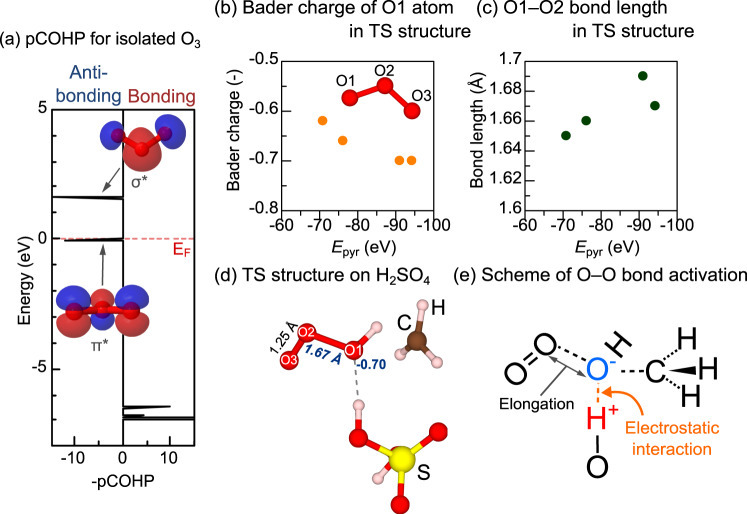
Theoretical interpretation of activation mechanism for CH_4_ + O_3_ reaction by acid sites. **a** The projected crystal orbital Hamilton population (pCOHP) curve for the O–O bond in the isolated O_3_ molecule. **b** Bader charge of O1 atom and **c** O1–O2 bond length in TS structures against *E*_pyr_ (shown in supplementary Fig. S8). **d** TS structure of CH_4_ + O_3_ reaction over H_2_SO_4_ molecule. Bader charge of O1 atom and bond lengths are shown together. **e** The schematic description of O–O bond activation scheme.

To quantitatively evaluate the impact of the acid property, the *E*_*a*_ of the CH_4_ + O_3_ reaction are plotted as a function of the stabilization energy of C_5_H_5_N on BASs (*E*_pyr_), as determined by DFT calculations. Notably, the adsorption of C_5_H_5_N on BAS of solid material has been widely applied to experimentally and theoretically analyze its acidity^60–62^. The values of *E*_pyr_ for HClO_4_, H_2_SO_4_, HNO_3_, and H_3_PO_4_ are −94.3, −91.1, −70.9, and −76.2 kJ/mol, respectively. This result corresponds to their experimentally obtained deprotonation enthalpies (see Supplementary Table S1). Figure 4d shows that the *E*_*a*_ of the CH_4_ + O_3_ reaction on BASs decreased with the increasing acid strengths, indicating that relatively strong BASs correspond to higher reaction rates for the CH_4_ + O_3_ reaction. To understand the activation mechanism within the frontier orbital theory, the projected crystal orbital Hamilton population (pCOHP) of an isolated O_3_ molecule was analyzed and described in Fig. 5a. There are two anti-bonding orbitals around the Fermi level (*E*_F_) in the pCOHP curve of O–O bond. In the visualization of their orbitals, lower and higher energies show π* and σ* characters, respectively. Since they have anti-bonding character, it is expected that more electron charge transfer to them induces weakening of the O–O bond. Figure 5b, c shows the Bader charge of O1 atom and the bond length of O1–O2 bond, respectively, against *E*_pyr_ in TS structure of CH_4_ + O_3_ reaction (the representative TS structure is shown in Fig. 5d and others are shown in supplementary Fig. S8). By increasing *E*_pyr_, the charge of O1 atom increases while O1–O2 bond is elongated, indicating that the O_3_ molecule was more activated on the acids with higher *E*_pyr_. A schematic description of the activation scheme is shown in Fig. 5e. By the electrostatic interaction between BAS and O1 atom, the charge transfer to O1 atom occurs, resulting in the elongation of O1–O2 bond, and thus, the BASs with high *E*_pyr_ efficiently decrease the *E*_*a*_ of CH_4_ + O_3_ reaction.

Inspired by the gained insight into BASs as active sites, a proton-type zeolite (ß-type) was theoretically examined for the adsorption of C_5_H_5_N (Fig. 4a, b). The result indicates the higher *E*_pyr_ of the zeolite (*E*_pyr_ = −199.8 kJ/mol) than that of H_2_SO_4_ (−91.1 kJ/mol). Note that *E*_pyr_ reflects not only the strength of acidity but also the confinement effect of the zeolite cage that also probably affects the CH_4_ combustion reaction^63,64^. The extrapolation of the linear relationship between *E*_pyr_ and *E*_*a*_ of the mineral acids indicates that the zeolite might achieve an extremely low *E*_*a*_ (~60 kJ/mol). To verify this hypothesis, we conducted TS calculations for the CH_4_ + O_3_ reaction on the BAS of the zeolite. Figure 4c shows the optimized structures of IS, TS, and FS. As expected from the strong acidity of the zeolite, the computed *E*_*a*_ (71.2 kJ/mol) is considerably lower than those for the mineral acids (126.2–142.2 kJ/mol).

Next, we experimentally verified the theoretically predicted relationship between the *E*_*a*_ and *E*_pyr_ of the CH_4_ + O_3_ reaction. The mineral acid (3 wt% H_2_SO_4_, HClO_4_, HNO_3_, or H_3_PO_4_) was loaded onto an SiO_2_ support and tested for the reaction in a 0.1% CH_4_ + 0.7% O_3_ flow (total flow: 100 ml/min, He balance) at 250 °C employing a fixed-bed flow reactor (the volatility of the acid was examined by H_2_O on-off switching test; Fig. S10). The concentrations of CH_4_ and O_3_ in the outlet gas were monitored by a gas cell that was equipped with an infrared spectroscope (Supplementary Fig. S9 shows the illustration of the experimental setup). Their CH_4_ conversion was maintained for 30 min. The obtained CH_4_ consumption rates are plotted as a function of *E*_pyr_ (Fig. 4e). Interestingly, the observed consumption rates correlate moderately with *E*_pyr_. Next, an Hß zeolite with a relatively low Si/Al ratio (8.5) (Hß8.5) was tested via the same reaction. Therein, Hß8.5 achieves an extremely higher consumption rate than H_2_SO_4_, demonstrating the highest rate among the tested catalysts. The above results demonstrate that the high CH_4_-combustion activity of a strong BAS-based catalyst, Hß8.5, could be rationally predicted based on computational mapping of the reaction network, as well as TS calculations. In the next section (Performance of the Hß-catalyzed CH_4_ combustion with O_3_), we experimentally demonstrate the superior performance of Hß8.5 by comparing it with Pd5Al_2_O_3_ as a conventional catalyst.

### Performance of the Hß-catalyzed CH_4_ combustion with O_3_

**Fig. 6:**
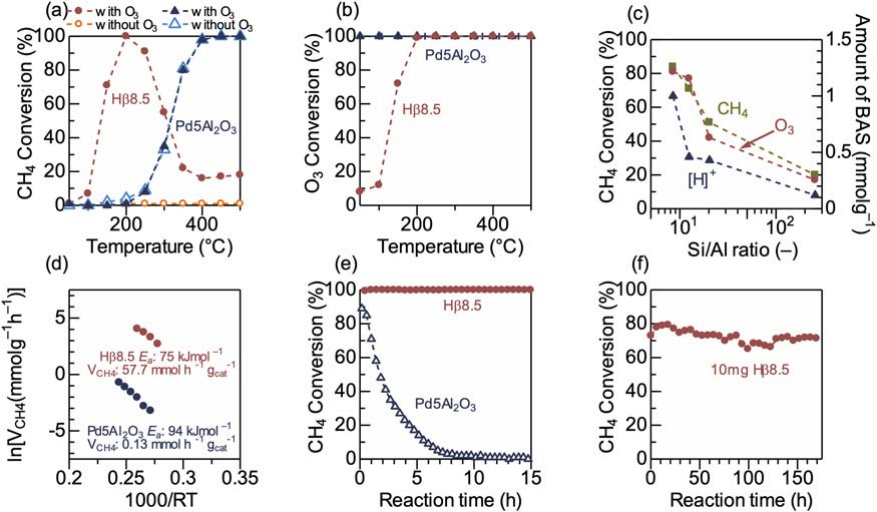
Catalytic test for CH_4_ combustion reaction. **a** CH_4_ and **b** O_3_ conversions over 40 mg of the Hß zeolite with an Si/Al ratio of 8.5 (Hß8.5) and 40 mg of 5 wt% Pd-loaded Al_2_O_3_ (Pd5Al_2_O_3_) in 0.1% CH_4_ + 5.95% O_2_ + 0.7% O_3_ and 0.1% CH_4_ + 10% O_2_ flows as functions of the reaction temperature. **c** Conversions of CH_4_ and O_3_ over 40 mg of the Hß zeolite with different Si/Al ratios (8.5, 12.5, 20, and 255) in a 0.1% CH_4_ + 5.95% O_2_ + 0.7% O_3_ flow at 150 °C, together with the amount of BAS in the ß zeolite, as evaluated via NH_3_-adsorption measurements. **d** Arrhenius plots for the combustions of CH_4_ over Hß8.5 in 0.1% CH_4_ + 5.95% O_2_ + 0.7% O_3_ at 160–190 °C, as well as over Pd5Al_2_O_3_ in 0.1% CH_4_ + 10% O_2_ at 170–220 °C. The reaction rates (V_CH4_) at 190 °C over Hß8.5 and Pd5Al_2_O_3_ are shown together (*R*^2^ = 0.99 for both catalyst). **e** Time course of CH_4_ conversion over 40 mg of Pd5Al_2_O_3_ (at 400 °C) and Hß8.5 (at 200 °C) in 0.1% CH_4_ + 3% H_2_O + 40 ppm SO_2_ + 10% O_2_ or 5.95% O_2_ + 0.7% O_3_ for Pd5Al_2_O_3_ and Hß8.5, respectively, with He balance (total flow: 100 ml/min). **f** Long-term reaction test for 10 mg of Hß8.5 in 0.1% CH_4_ + 5.95% O_2_ + 0.7% O_3_ + 3% H_2_O + 40 ppm SO_2_ at 200 °C.

We conducted catalytic tests to experimentally demonstrate the catalytic performance of Hß8.5. Figure 6a shows the conversions of CH_4_ over Hß8.5 and a benchmark catalyst (Pd5Al_2_O_3_) in a flow of 0.1% CH_4_ + 5.95% O_2_ + 0.7% O_3_ at different temperatures. In 0.1% CH_4_ + 10% O_2_, Hß8.5 did not achieve CH_4_ conversion in the entire temperature range, indicating the necessity of O_3_ as the oxidant. In 0.1% CH_4_ + 5.95% O_2_ + 0.7% O_3_, Hß8.5 achieved the high conversion of CH_4_ at 200 °C, while Pd5Al_2_O_3_ required a temperature of >400 °C to achieve comparable performance. At >250 °C, the conversion of CH_4_ over Hß8.5 decreased because O_3_ conversion had reached 100% via self-decomposition (Fig. 5b). To evaluate the effect of Pd loading into zeolites, Pd-exchanged ß8.5 zeolite (Pdß8.5) was prepared and then tested, resulting in low CH_4_ conversion in the low-temperature region due to full decomposition of O_3_ similar to the result of Pd5Al_2_O_3_ (Supplementally Fig. S11). The effect of BAS on the self-decomposition of O_3_ into O_2_ (2O_3_ → 3O_2_) was theoretically investigated because this reaction represented an obstacle to the practical application of O_3_ as an oxidant. Supplementary Fig. S12 compares the optimized TS structures of the uncatalyzed and Hß-catalyzed decompositions of O_3_. The calculated *E*_*a*_ of the uncatalyzed and Hß-catalyzed reactions are 42.3 and 69.5 kJ/mol, respectively, indicating that BAS in zeolite do not promote the self-decomposition of O_3_. This property is desirable in catalysts for CH_4_ combustion by O_3_. Dissimilar to Hß8.5, Pd5Al_2_O_3_ exhibited high activity toward O_3_ decomposition (complete conversion even at 50 °C); hence, the addition of O_3_ did not increase the CH_4_ combustion activity of Pd5Al_2_O_3_.

Figure 5c shows a plot of the conversions of CH_4_ and O_3_, as well as the BAS amounts, as evaluated by NH_3_-adsorption measurement as a function of the Si/Al ratio of the utilized ß zeolites (Si/Al = 8.5, 12.5, 20, and 255). Evidently, the Al-rich ß zeolites with more BASs exhibited higher CH_4_ conversions, which is consistent with the proposal from DFT calculation above. The Arrhenius plot of the Hß8.5-catalyzed CH_4_ combustion with O_3_ (Fig. 5d) revealed an apparent barrier (*E*a) of 75 kJ/mol, which agrees with the theoretical value of the CH_4_ + O_3_ reaction to yield CH_3_OH (*E*_a_ = 71.2 kJ/mol). The *E*_a_ of Hß8.5 was considerably lower than that of Pd5Al_2_O_3_ (*E*_a_ = 95 kJ/mol) in 0.1% CH_4_ + 10% O_2_. Supplementary Fig. S13 shows the results of the kinetic analyses for estimating the reaction orders. The reaction orders of CH_4_ (0.2) and O_3_ (0.2) were positive. Further, the reaction rates per weight of the catalyst (for Hß8.5 and Pd5Al_2_O_3_) for CH_4_ conversion (*V*_CH4_) at 190 °C were compared, and the result indicated that *V*_CH4_ of Hß8.5 (57.5 mmol h^−1^ g_cat_^−1^) was 442 times higher than that of Pd5Al_2_O_3_ (0.13 mmol h^−1^ g_cat_^−1^), demonstrating that the found main-group catalyst (Hß8.5) exhibited considerably higher activity toward CH_4_ combustion than the PGM-based benchmark catalyst (scientific literature on the same reaction using proton-type zeolite are limited, and thus, it is difficult to compare with the previous reports.).

Further, Hß8.5 and Pd5Al_2_O_3_ were tested for CH_4_ combustion in the co-presence of H_2_O and SO_2_ to compare their resistance to steam and SOx poisoning. Figure 5f shows the time course of CH_4_ combustion over 40 mg of Hß8.5 (5.95% O_2_ + 0.7% O_3_ at 200 °C) and Pd5Al_2_O_3_ (10% O_2_ at 400 °C) in 0.1% CH_4_ + 3% H_2_O + 40 ppm SO_2_. By feeding H_2_O and SO_2_, the CH_4_ conversion at 400 °C over Pd5Al_2_O_3_ decreased with the reaction time, reaching almost zero after 10 h. Conversely, the CH_4_ conversion over Hß8.5 did not decrease at 200 °C even after 15 h, indicating that Hß8.5 was highly resistant to steam and SO_2_ poisoning. Finally, 10 mg of Hß8.5 was examined for the long-term reaction test in 0.1% CH_4_ + 5.95% O_2_ + 0.7% O_3_ + 3% H_2_O + 40 ppm SO_2_ at 200 °C, and the result indicated that the catalyst did not significantly decrease the CH_4_ conversion for 170 h of reaction time.

To investigate the mechanism of the catalytic CH_4_ combustion by O_3_ in the realistic condition, ab initio molecular dynamics (AIMD) was additionally carried out for CH_4_ and O_3_ separately. During the simulation, the O_3_ molecule frequently interacted with BASs and was present near the sites. In contrast, the CH_4_ molecule diffused throughout the zeolite cage and less frequently interacted with BASs. In fact, the calculated mean diffusion coefficient of the O_3_ molecule is 5.0 × 10^−4^ Å^2^/fs in the simulation, while that of the CH_4_ molecule is 8.4 × 10^−4^ Å^2^/fs. These results indicate that, in realistic conditions, the O_3_ molecule is present near BASs, and once CH_4_ reaches the sites, CH_4_ and O_3_ react to form CH_3_OH and O_2_.

## Discussion

In this study, we rationally designed a catalyst for low-temperature O_3_-driven catalytic combustion of CH_4_ based on the elucidation of an unexplored reaction network. The CH_4_ + O_3_ reaction toward generating CO_2_ was explored via SC-AFIR, and the formation of CH_3_OH via CH_4_ + O_3_ (CH_4_ + O_3_ → CH_3_OH + O_2_) was determined as RDS of CH_4_ combustion (*E*_*a*_ = 142.7 kJ/mol). To assess the various types of active sites (acid, base, redox, and radical sites), model molecules (H_2_SO_4_, C_5_H_5_N, a Cu atom, and HNO_3_) were introduced into the system, and reaction route was calculated. Among the examined active sites, H_2_SO_4_ effectively decreased the reaction (*E*_*a*_ = 126.2 kJ/mol); thus, different Brønsted acid catalysts with different acid strengths were examined for the TS calculation. The relationship between the acidity and calculated *E*_*a*_ of the CH_4_ + O_3_ reaction (CH_3_OH formation) availed a facile catalyst design concept; the stronger the BASs afford the higher the catalytic activity. Thereafter, the theory-driven concept was experimentally verified by CH_4_ combustion with O_3_ at 250 °C. An Hß zeolite, which was the most effective candidate, as predicted by this concept, was experimentally tested for the CH_4_ + O_3_ reaction. The apparent activation energy (75 kJ/mol), which was estimated by the kinetic experiment, was consistent with the computed value (71.2 kJ/mol). Hß exhibited a very high reaction rate, which was 442 times higher than that of the benchmark catalyst, Pd5Al_2_O_3_, at 190 °C. During the catalytic tests in the presence of SO_2_ and H_2_O, Hß achieved the full conversion of CH_4_ at 190 °C, whereas Pd5Al_2_O_3_ was completely deactivated even at a higher temperature (400 °C) owing to the poisoning of its active sites by water and SO_2_. Finally, the developed catalyst (Hß zeolite) was tested in a 170-h long-term reaction that exhibited very high resistance against water and SO_2_.

In summary, O_3_, which was determined as an efficient oxidant, oxidized CH_4_ into CH_3_OH as the RDS. Among the considered active sites, BAS in H_2_SO_4_ molecule efficiently decreased the activation barrier of CH_3_OH formation. After stronger BASs were evaluated to be promising theoretically and experimentally, proton-type zeolite, comprising strong BASs, was experimentally tested; Hß zeolite exhibited the superior CH_4_ combustion rate in the presence of O_3_. These results demonstrated that a computationally designed catalysis based on earth-abundant metal elements (Si and Al) and alternative oxidants could achieve higher activities and durabilities compared with their PGM-based ones for low-temperature CH_4_ combustion.

## Methods

### DFT calculations

Spin-polarized DFT calculations were performed employing the generalized-gradient approximation of Perdew–Burke–Ernzerhof functional^65^, as implemented in the Vienna Ab Initio Simulation Package^66,67^ (VASP), and the projected augmented waves^68,69^ method was employed for the Kohn–Sham equations with cut-off energy of 500 eV. The Γ point was employed for the Brillouin-zone sampling^70^. DFT-D3 dispersion correction with the Becke–Johnson damping was employed for all the calculations^71^. To simulate the gas-phase reaction, calculations were conducted within a large cubic cell (*a* = *b* = *c* = 15 Å). The structure of the β zeolite was obtained from the International Zeolite Association database^72^, and the lattice constants were fixed at initial values (*a* = *b* = 12.632 Å, *c* = 26.186 Å, *α* = *β* = *γ* = 90.0°) during the calculations. The SC-AFIR method, as implemented in the GRRM17 program^38^, was applied for reaction route mapping with a model collision energy parameter of 1000 kJ/mol. Only a positive force was applied for the AFIR calculations. The H, O, and C atoms in the CH_4_ and O_3_ molecules were considered the targets of SC-AFIR. The locally updated plane method availed the path top points, which were subsequently optimized as TS structures and determined by the following intrinsic reaction coordinate calculation^73^. To calculate for the β zeolite, the atoms of the zeolitic framework, except for the Al atom, as well as the Si atoms adjacent to the Al and O atoms connecting to the adjacent Si, and H atoms of BAS were fixed at the crystallographic position (Fig. 4b). The stabilization energy of C_5_H_5_N was defined, as follows:

1 $${E}_{{{{{{\rm{pyr}}}}}}}=({E}_{{{{{{\rm{C}}}}}}5{{{{{\rm{H}}}}}}5{{{{{\rm{N}}}}}}\; {{{{{\rm{on}}}}}}\; {{{{{\rm{BAS}}}}}}}-{E}_{{{{{{\rm{BAS}}}}}}}-{E}_{{{{{{\rm{C}}}}}}5{{{{{\rm{H}}}}}}5{{{{{\rm{N}}}}}}})$$

Thus, the total energies of the models including a C_5_H_5_N molecule, acid molecules, and C_5_H_5_N interacting with the acid molecules were described as *E*_C5H5N_, *E*_BAS_, and *E*_C5H5N on BAS_, respectively. The pCOHP^74^ was calculated by LOBSTER software^75^. The molecular orbitals were visualized with the VASPMO code^76^. The structures are visualized by VESTA software^77^. AIMD simulation was performed within the periodic-boundary condition as implemented in CP2K package^78^. The spin-unrestricted DFT calculation was carried out using the Perdew–Burke–Ernzerhof functional^65^ within the generalized-gradient approximation. The valence electrons were described by the double-ζ valence plus polarization basis sets of the MOLOPT type^79^, and the core electrons were represented by the Goedecker–Teter–Hutter pseudopotentials^80,81^. The energy cutoff was set to 500 Ry. Only the Γ-point was employed for the Brillouin zone integration. The Born–Oppenheimer MD simulation was carried out with the canonical (NVT) ensemble condition using the Nóse−Hoover thermostat to control the temperature. The time step was set to 1 fs. After equilibrating for 1 ps, 50 ps of the simulation was performed for each model comprising one CH_4_ or O_3_ molecule, respectively.

### Catalysts preparations

1 g of proton-type (H) β zeolite with a Si/Al ratio of 8.5 (Hβ 8.5) was obtained via the calcination of an NH_4_^+^-type β zeolite that was purchased from Tosoh Co. (HSZ-920NHA) in the air at 500 °C. The 1 g of Hβ zeolites with Si/Al ratios of 20 and 255 (HSZ-940HOA and HSZ-980HOA, respectively) were supplied by Tosoh Co., and another with a Si/Al ratio of 12.5 was supplied by the Catalysis Society of Japan (JRC-Z-HΒ25). Note that the impurity of Hβ8.5 was checked by ICP measurement, and Fe, Co, Ni, Cu, and Zn were not detected (<0.01 wt%). Acid-load SiO_2_ was prepared via impregnation method. 0.2 g of SiO_2_ (CARiACT G–6, Fuji Silysia, *S*_BET_ = ca. 500 m^2^ g^–1^) was suspended in an 0.5 M aqueous solution of H_2_SO_4_, HClO_4_, H_3_PO_4_, and HNO_3_ at room temperature. The water was evaporated at 50 °C from the mixture and dried in an oven at 90 °C. 1 g of Al_2_O_3_ was prepared by calcining γ-AlOOH (Catapal B Alumina, Sasol) for 3 h at 900 °C. Next, 1.05 g of 5 wt% Pd-loaded Al_2_O_3_ was prepared by impregnating 1 g of Al_2_O_3_ with an 0.005 M aqueous HNO_3_ solution Pd(NH_3_)_2_(NO_3_)_2_. 0.5 g of Pd-exchanged ß zeolite (Pdß8.5) was prepared by exchanging NH_4_^+^− ß8.5 with an 0.005 M aqueous solution of [Pd(NH_3_)_4_]Cl_2_ at room temperature for 20 h, followed by centrifuging and washing with deionized water, drying (100 °C, 20 h), and by calcining (500 °C, 1 h, in the air).

### Catalytic test

The catalytic test was conducted under in 0.1% CH_4_ + 0.7% O_3_ + 5.95% O_2_ (He balance, total flow:100 ml/min) as a typical condition. 40 mg of catalyst powder was set in the fixed-bed reactor using quartz wool and the outlet was directly connected to a JASCO FT/IR-4600 spectrometer that was equipped with a triglycine sulfate (TGS) detector, in which a homemade infrared (IR) gas cell, which was equipped with KBr windows, was placed to monitor the concentrations of CH_4_ and O_3_. For kinetic analysis, 5 mg of Hβ 8.5 was used to keep CH_4_ conversion under 40%. The IR area of O_3_ was calibrated employing an O_3_ analyzer (EG-550, EcoDesign Inc.) to convert the area into concentration. To feed O_3_ into the system, an O_2_ flow was passed through an ozonizer (F0G-AC5G, EcoDesign Inc.) that was placed before the mainstream. The whole view of the employed setup is shown in Supplementary Fig. S9. The NH_3_-adsorption measurement was carried out by Infrared spectroscopy (JASCO FT/IR-4200 spectrometer using a home-made in situ IR cell. The catalyst disc of the zeolite sample (40 mg,) was dehydrated under He flow at 500 °C before a background spectrum was recorded under a flow of He at 200 °C. Then, NH_3_ (1%) flowed to the sample, followed by He purging before IR spectrum was taken at 200 °C.

### Statistics and reproducibility

The experiments were not randomized.

## Supplementary information

Supplementary Information

## Data Availability

The source data, which support the result of this study, can be found in the manuscript and Supplementary information. Data are available from the corresponding author upon request.

## Code availability

The VASP code package used in this work can be accessible after a user license is authorized by the VASP company (https://www.vasp.at). LOBSTER, VASPMO, VESTA, and CP2K codes can be freely accessible from the websites (LOBSTER: http://www.cohp.de, VASPMO: https://sourceforge.net/projects/vaspmo/, VESTA: https://jp-minerals.org/vesta/en/, and CP2K: https://www.cp2k.org). AIMD simulation was carried out using Quickstep module in CP2K code.
