# Supplementary material for: Designing main-group catalysts for low-temperature methane combustion by ozone
Source: Nat Commun. 2023 Jul 3;14:3926. doi: 10.1038/s41467-023-39541-y (PMC10317982; doi:10.1038/s41467-023-39541-y)
Supplement: Supplementary file 1 — Supplementary Information [file 41467_2023_39541_MOESM1_ESM.pdf]

Designing Main-Group Catalysts for Low-Temperature Methane Combustion by Ozone

Shunsaku Yasumura,[1] Kenichiro Saita,[2] Takumi Miyakage[1], Ken Nagai[1], Kenichi Kon[1], Takashi Toyao,[1] Zen Maeno,[3] Tetsuya Taketsugu,[2,4] Ken-ichi Shimizu*,[1]

[1] Institute for Catalysis, Hokkaido University, N-21, W-10, Sapporo, Hokkaido 001-0021, Japan

[2] Department of Chemistry, Faculty of Science, Hokkaido University, Sapporo, Hokkaido 060-0810, Japan

[3] School of Advanced Engineering, Kogakuin University, Tokyo, 192-0015, Japan

[4] Institute for Chemical Reaction Design and Discovery (WPI-ICReDD), Hokkaido University, Sapporo, Hokkaido 001-0021, Japan

Table and Figures

Table S1 Summarized data of acids for stabilization energy of pyridine on the BASs (E_{pyr}) and reported experimental value of deprotonation enthalpy (ΔH_{DP}).

Acids	E_{pyr} (kJ/mol)	ΔH_{DP} (kJ/mol)
H_3PO_4	-76.2	1383 ± 21[1]
HNO_3	-70.9	1358 ± 0.84[2]
H_2SO_4	-91.1	1295 ± 11[3]
$HClO_4$	-94.3	1255 ± 24[4]

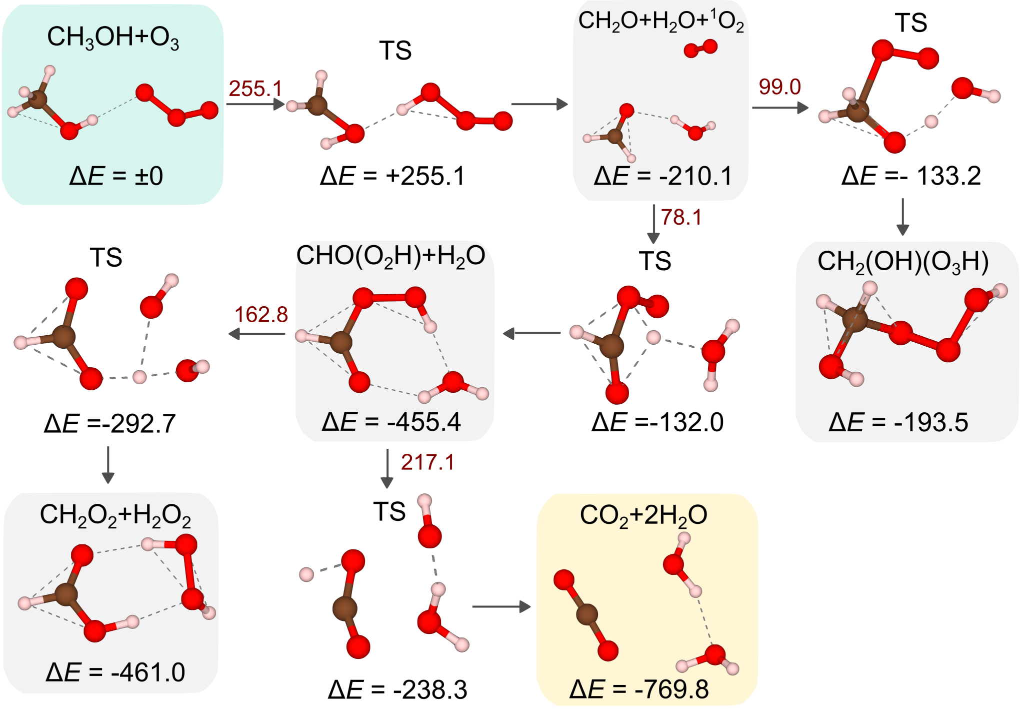

Fig. S1 Calculated reaction pathway of $CH_3OH + O_3$ together with the values of relative energy (ΔE). Values written in dark red shows the activation barrier. (Unit: kJ/mol)

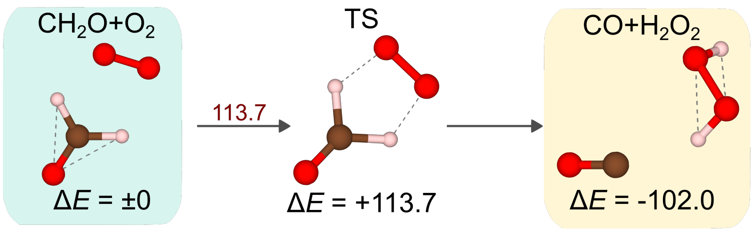

Fig. S2 Calculated reaction pathway of CH_2O + O_2 together with the values of relative energy (Δ*E*). Values written in dark red shows the activation barrier. (Unit: kJ/mol)

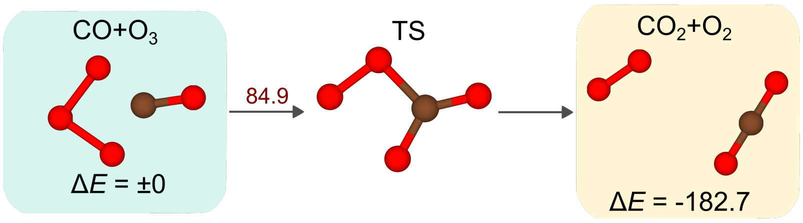

Fig. S3 Calculated reaction pathway of CO + O_3 together with the values of relative energy (Δ*E*). Values written in dark red shows the activation barrier. (Unit: kJ/mol)

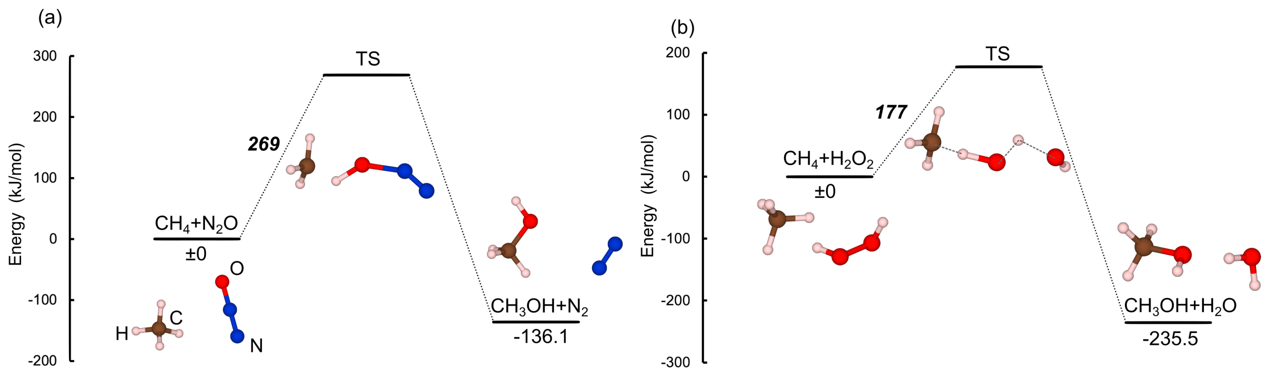

Fig. S4 Energy profiles of CH_3OH formation from (a) CH_4 + N_2O or (b) CH_4 + H_2O_2 reactions. Relative energies are provided under each bar and the activation barriers are described by bold italic style. (Unit: kJ/mol)

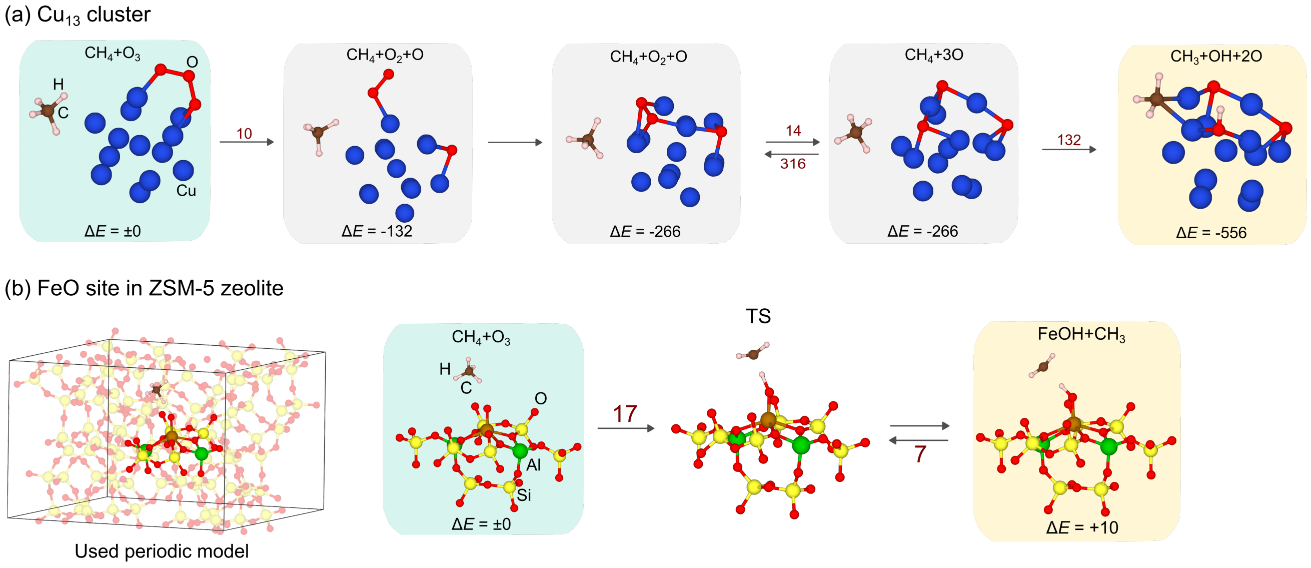

Fig. S5 Calculated reaction pathways of CH_4 + O_3 on (a) Cu_{13} cluster and (b) FeO species in ZSM-5 zeolite. The values of Δ*E* and the *Ea* (dark red) are shown together (Unit: kJ/mol).

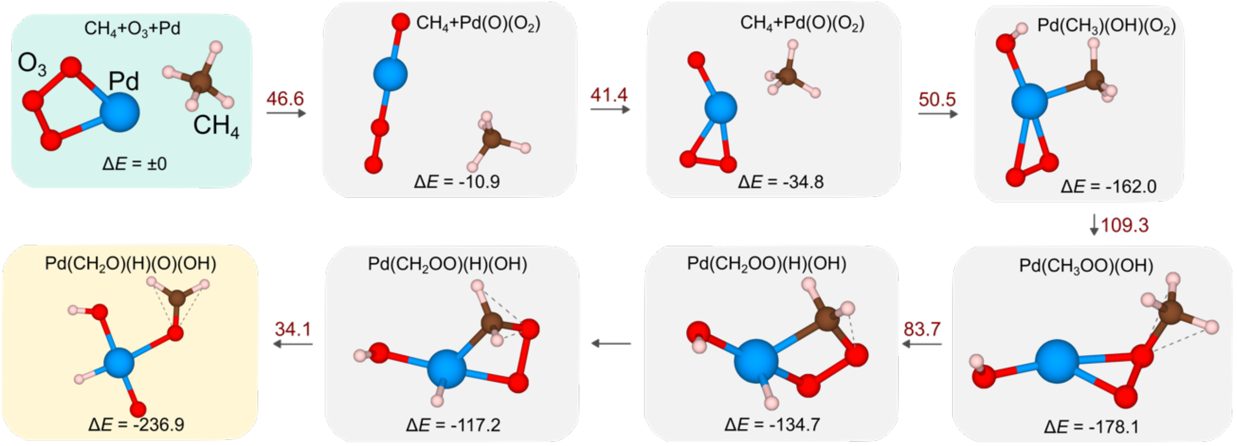

Fig. S6 Calculated reaction pathways of CH_4 + O_3 on Pd atom. The values of Δ*E* and the *Ea* (dark red) are shown together (Unit: kJ/mol).

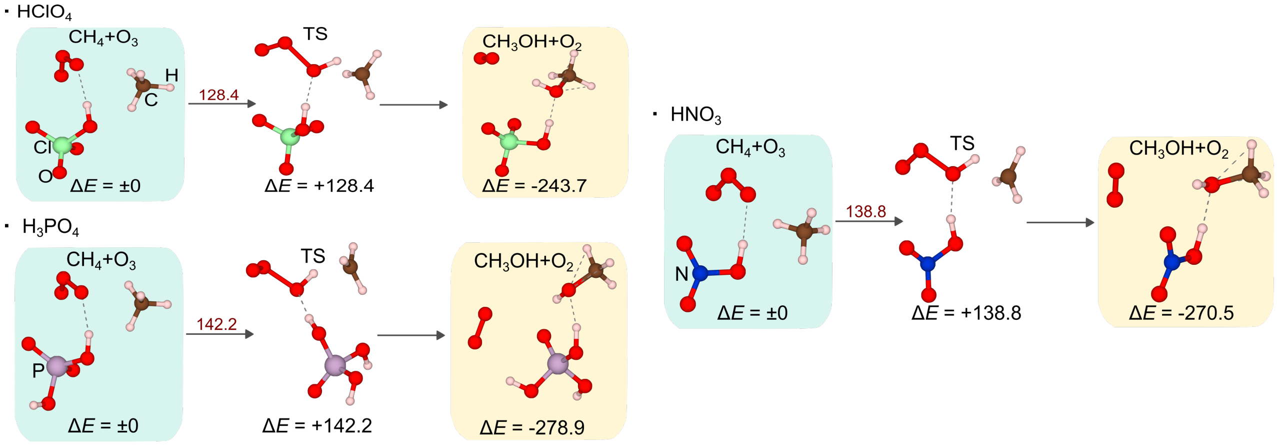

Fig. S7 Calculated reaction pathway of CH_4 + O_3 on $HClO_4$, H_3PO_4, and HNO_3. Relative energy (ΔE) is provided in the unit of kJ/mol. Activation barriers (*Ea*) are shown with dark red color.

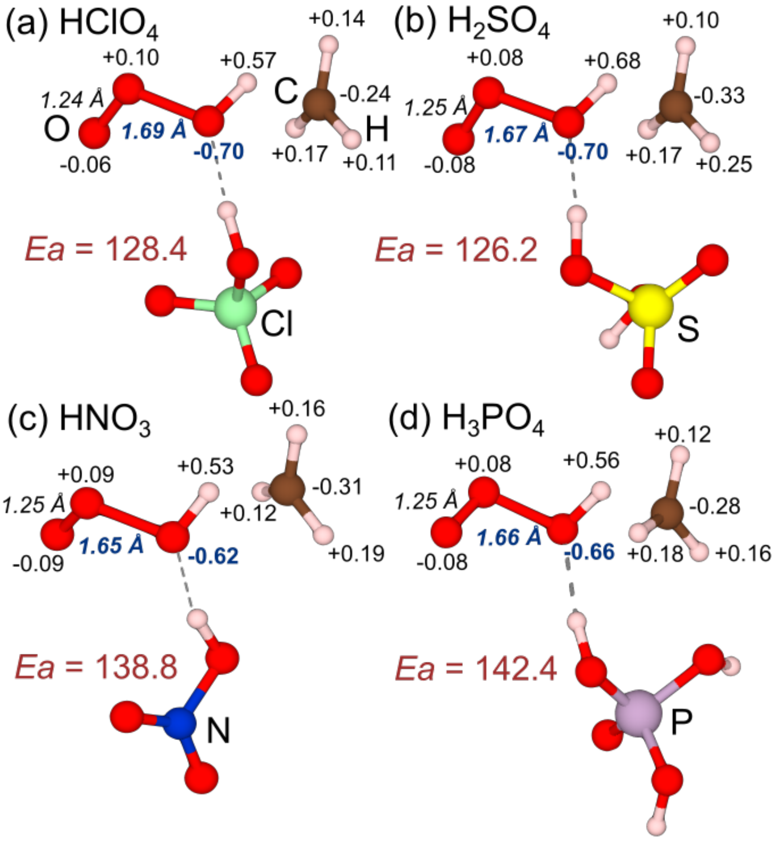

Fig. S8 Bader charge analysis of the structure of the transition state of CH_4 + O_3 reaction on (a) $HClO_4$, (b) H_2SO_4, (c) HNO_3, and (d) H_3PO_4.

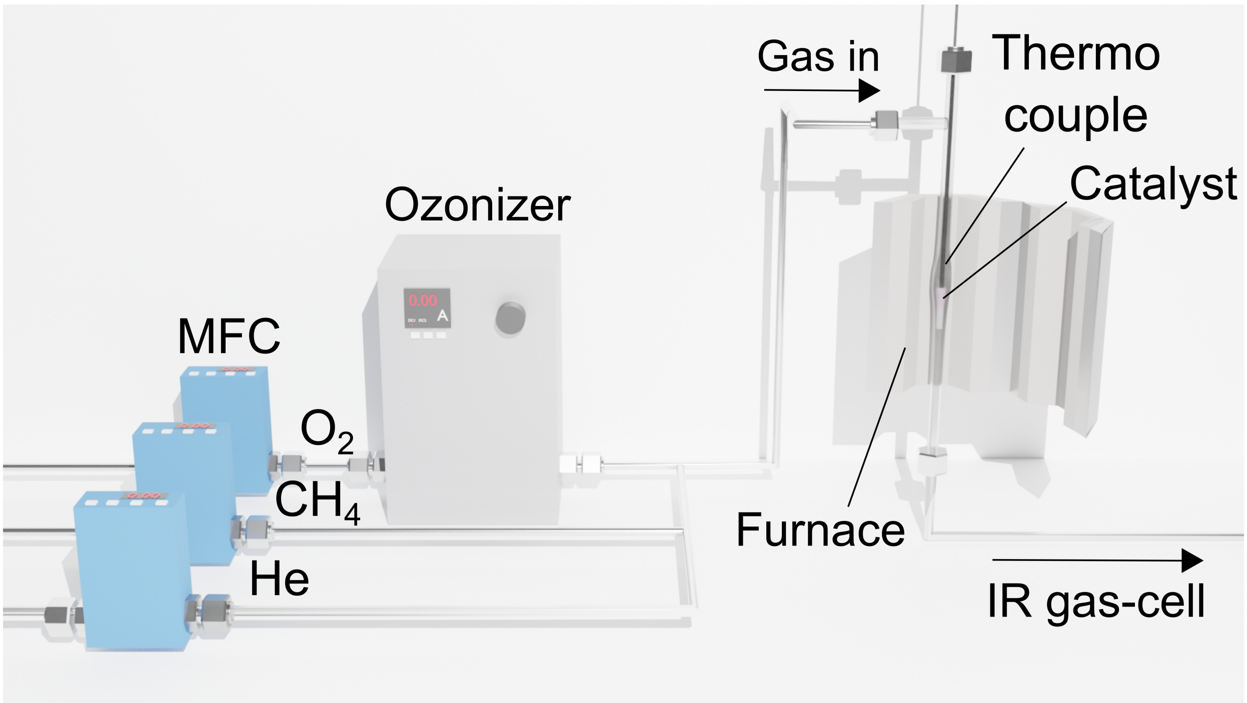

Fig. S9 Illustration of the experimental setup for CH_4 combustion test with ozonizer.

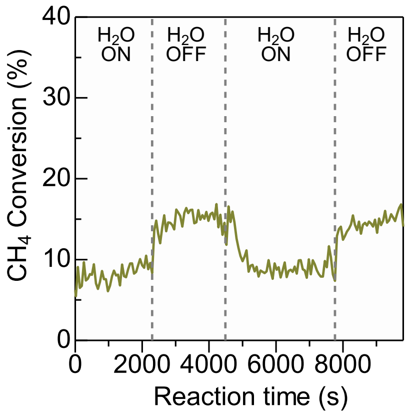

Fig. S10 CH_4 conversions over 40 mg of 3wt% H_2SO_4-loaded SiO_2 in 0.1% CH_4 + 0.7% O_3 (with/without 3% H_2O feeding) flow at 250 ˚C

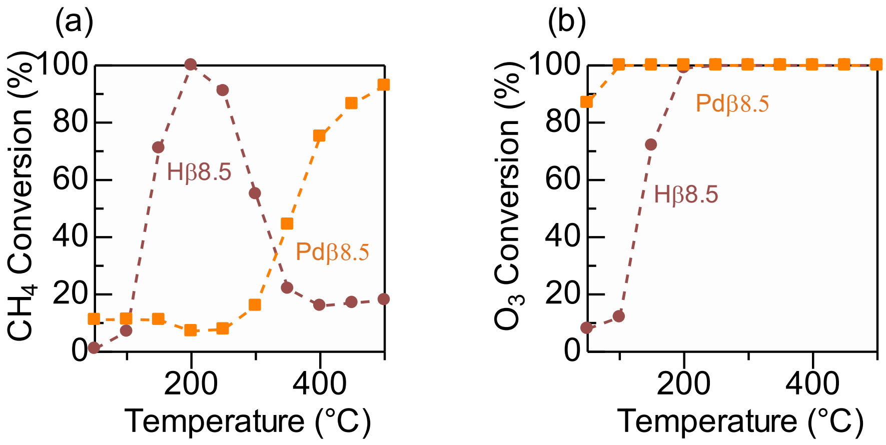

Fig. S11 (a) CH_4 and (b) O_3 conversions over 40 mg of the Hß zeolite with a Si/Al ratio of 8.5 (Hß8.5) and 40 mg of Pd-exchanged ß zeolite (Pd/Al = 0.05; Pdß8.5) in 0.1% CH_4 + 0.7% O_3 flow as functions of the reaction temperature.

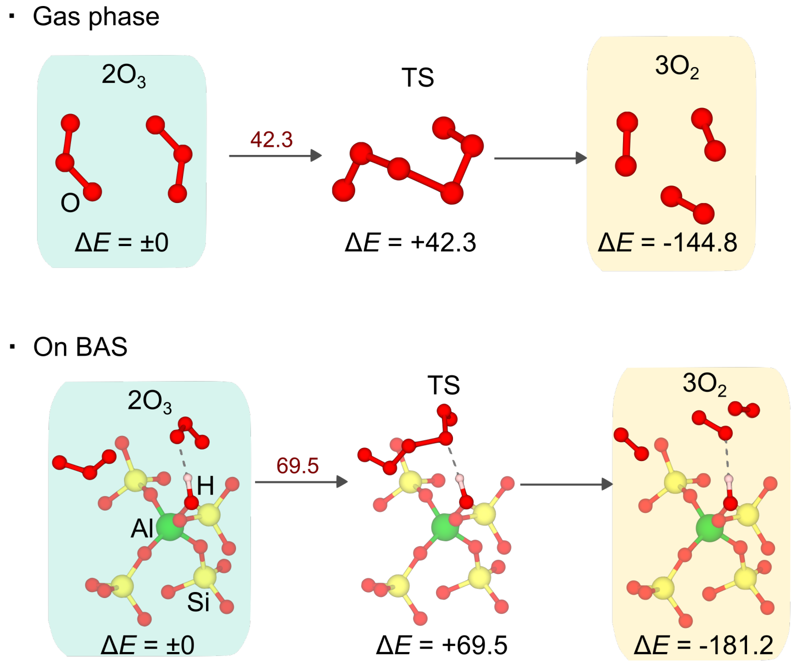

Fig. S12 Transition state calculations for self-decomposition reaction of ozone in gas-phase and on Brønsted acid site (BAS) of BEA zeolite. Relative energy (ΔE) is provided in the unit of kJ/mol. Activation barriers (*Ea*) are shown with dark red color. Only atoms around BAS are shown for cleairity.

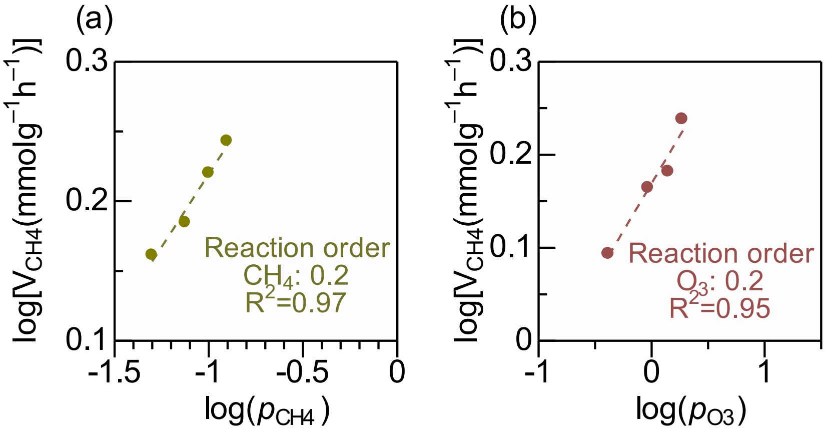

Fig. S13 Log−log plot of the reaction rate of CH_4 consumption at 130 °C vs (a) CH_4 pressure (at 0.7% O_2) and (b) O_3 pressure (at 0.1% CH_4)

Supplementary References

1. Morris, R. A., Knighton, W. B., Viggiano, A. A., Hoffman, B. C. & Schaefer III, H. F. The gas-phase acidity of H_3PO_4. *The Journal of Chemical Physics* **106**, 3545–3547 (1997).
2. Mathur, B. P., Rothe, E. W., Tang, S. Y., Mahajan, K. & Reck, G. P. Negative gaseous ions from nitric acid. *The Journal of Chemical Physics* **64**, 1247–1248 (1976).
3. Viggiano, A. A., Henchman, M. J., Dale, F., Deakyne, C. A. & Paulson, J. F. Gas-Phase Reactions of Weak Bronsted Bases I^-, PO_3^-, HSO_4^-, FSO_3^-, and $CF_3SO_3^-$ with Strong Bronsted Acids H_2SO_4, FSO_3-H, and CF_3SO_3H. A Quantitative Intrinsic Superacidity Scale for the Sulfonic Acids XSO_3H (X = HO, F, and CF_3). 4299–4306 (1992).
4. Meyer, M. M. & Kass, S. R. Experimental and theoretical gas-phase acidities, bond dissociation energies, and heats of formation of $HClO_x$, x = 1-4. *Journal of Physical Chemistry A* **114**, 4086–4092 (2010).
